# Computational analysis of the interaction between transcription factors and the predicted secreted proteome of the yeast *Kluyveromyces lactis*

**DOI:** 10.1186/1471-2105-10-194

**Published:** 2009-06-25

**Authors:** Otávio JB Brustolini, Luciano G Fietto, Cosme D Cruz, Flávia ML Passos

**Affiliations:** 1 Departamento de Microbiologia, Instituto de Biotecnologia Aplicada à Agropecuária (BIOAGRO), Universidade Federal de Viçosa, Viçosa, MG, Brazil; 2 Departamento de Bioquímica e Biologia Molecular, Instituto de Biotecnologia Aplicada à Agropecuária (BIOAGRO), Universidade Federal de Viçosa,Viçosa, MG, Brazil; 3 Departamento de Biologia Geral, Instituto de Biotecnologia Aplicada à Agropecuária (BIOAGRO), Universidade Federal de Viçosa, Viçosa, MG, Brazil

## Abstract

**Background:**

Protein secretion is a cell translocation process of major biological and technological significance. The secretion and downstream processing of proteins by recombinant cells is of great commercial interest. The yeast *Kluyveromyces lactis *is considered a promising host for heterologous protein production. Because yeasts naturally do not secrete as many proteins as filamentous fungi, they can produce secreted recombinant proteins with few contaminants in the medium. An ideal system to address the secretion of a desired protein could be exploited among the native proteins in certain physiological conditions. By applying algorithms to the completed *K. lactis *genome sequence, such a system could be selected. To this end, we predicted protein subcellular locations and correlated the resulting extracellular secretome with the transcription factors that modulate the cellular response to a particular environmental stimulus.

**Results:**

To explore the potential *Kluyveromyces lactis *extracellular secretome, four computational prediction algorithms were applied to 5076 predicted *K. lactis *proteins from the genome database. SignalP v3 identified 418 proteins with N-terminal signal peptides. From these 418 proteins, the Phobius algorithm predicted that 176 proteins have no transmembrane domains, and the big-PI Predictor identified 150 proteins as having no glycosylphosphatidylinositol (GPI) modification sites. WoLF PSORT predicted that the *K. lactis *secretome consists of 109 putative proteins, excluding subcellular targeting. The transcription regulators of the putative extracellular proteins were investigated by searching for DNA binding sites in their putative promoters. The conditions to favor expression were obtained by searching Gene Ontology terms and using graph theory.

**Conclusion:**

A public database of *K. lactis *secreted proteins and their transcription factors are presented. It consists of 109 ORFs and 23 transcription factors. A graph created from this database shows 134 nodes and 884 edges, suggesting a vast number of relationships to be validated experimentally. Most of the transcription factors are related to responses to stress such as drug, acid and heat resistance, as well as nitrogen limitation, and may be useful for inducing maximal expression of potential extracellular proteins.

## Background

The General Secretory Pathway (GSP) is a protein export process of major biological and technological significance. Cell communication, as well as intercellular signaling and growth during development in multicellular organisms depends on the secretion pathway. The export of a commercial protein into the extracellular medium by a recombinant cell can facilitate its downstream processing. The yeast *Kluyveromyces lactis *is considered a promising host for heterologous protein production. Because yeasts naturally do not secrete as many proteins as filamentous fungi, they can produce secreted recombinant proteins with few contaminants in the medium [1]. An ideal system for secreting a desired protein could be developed from analysis of the native proteins. The completed *K. lactis *genome sequence provides the tools to construct such a system [2]. As the genomes of several hemiascomycetes yeasts are now sequenced [3-5] and cross-comparison does not reveal significant differences, the prospect of discovering a potentially significant secreted protein using bioinformatics techniques is high [6-8]. In *K. lactis*, as in other eukaryotes, secreted proteins are typically recognized by the presence of an N-terminal signal sequence to direct them to GSP [11]. Signal sequences usually have a well-characterized structure composed of a central hydrophobic core (h-region). This consists of an average of 6–15 amino acid (aa) residues that are flanked by hydrophilic N- and C-terminal regions. The h-region is important for correct targeting and membrane insertion of the peptide. At the polar C-terminal region, helix breaking often occurs because of proline and glycine residues and small uncharged residues at the -3 and -1 positions that determine the signal peptide cleavage site [9,10]. The polar N-terminal region is variable in length and frequently positively charged [11]. Although some proteins lacking N-terminal signal sequences reach the extracellular medium, the majority of soluble secreted proteins in *K. lactis *are likely to be transported via the GSP [1]. A wide variety of computational methods have been used to predict the subcellular localization of proteins [12]. The methods differ in the input data they demand and the techniques applied to make decisions or predictions about location. Once the input data type are fixed, the methods for making predictions are basically by two methods: the manual construction of explicit rules for localization prediction using current knowledge of sorting signals, or applying data-driven, machine-learning techniques (e.g., Neural Networks (NN) or Hidden Markov Models, (HMMs)) [12]. The latter automatically extracts decision rules from the sets of proteins with known location, without making any prior, detailed assumptions about the features of interest.

In addition to using direct algorithm analysis to predict extracellular proteins, the extracellular secretome can be analyzed through its possible transcription factors (TFs). TFs are part of the signal transduction pathway that modulates the cell metabolism in response to environmental stimuli [13]. The TFs that contain DNA binding motifs are the component of the signaling pathway that is closest to the level of the DNA. To a large degree, the combinatorial presence and absence of transcription factor binding sites (TFBSs) is responsible for gene regulation complexity [14-17]. The identification of TFBSs has been used to infer regulatory networks for several different yeasts [18].

Using an algorithm approach, we proposed identifying extracellular protein candidates in the yeast *K. lactis *and determining TFBSs in the promoters of their genes. Analysis of the relationship to transcriptional regulators used the dataset of Bussereau *et al *[18], and putative promoter regions 1 kb upstream of the genes that encode the predicted extracellular proteins.

## Results

### Prediction of *K. lactis *extracellular proteins

**Figure 1:**
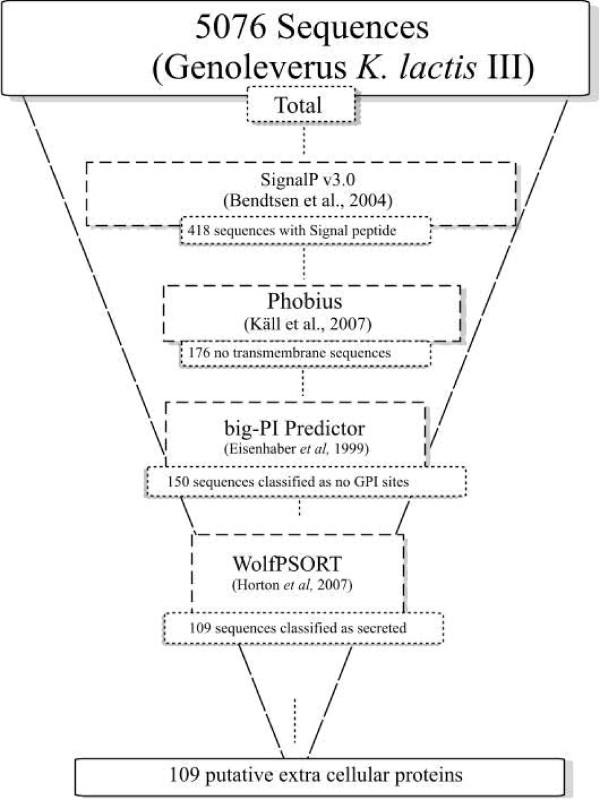
**Flowchart of the strategy adopted for mining *K. lactis *gene sequences for extracellular proteins and the outcome**.

A flowchart of the algorithms used to generate the database of potential extracellular *K. lactis *proteins is in Figure 1. Using the standard criteria of SignalP v3.0 [11], and the NN and HMM scores from 5076 *K. lactis *open reading frames (ORFs), 698 ORFs containing consensus sequences for N-terminal signal peptides and signal peptidase cleavage sites within 10–40 amino acid residues were predicted. When the 418 deduced proteins harboring N-terminal signal peptides were submitted to the Phobius algorithm [19], only 242 were predicted to carry extra transmembrane domains, excluding the transmembrane domain of the signal peptide. The following analyses were conducted with the remaining 176 ORFs. To identify GPI modification sites, the ORFs were submitted to big-PI Predictor [20], with the results indicating that 150 ORFs contained a signal peptide, no transmembrane domain, and no GPI modification site. As some GSP proteins may be targeted to intracellular organelles rather than the extracellular medium, the algorithm WoLF PSORT [21] was used to detect conserved addresses to organelles. The outcome indicated 150 ORFs predicted with extracellular addresses. Among these, 109 had the highest *k*-NN score (~17.78 ± 5.68)l the remaining 41 had lower *k*-NN scores (~5.354 ± 3.989) and were excluded, to increase the probability of selecting actual secreted proteins for further correlation with the transcription factor dataset.

**Figure 2:**
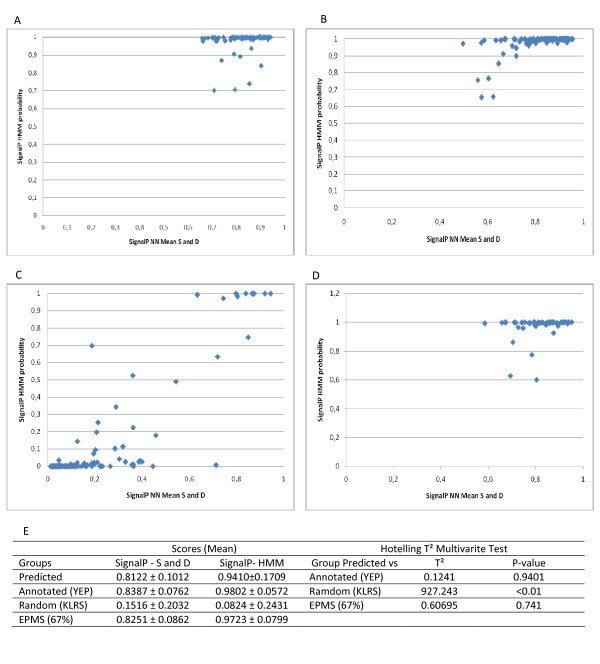
**Analysis of the distribution of SignalP v3.0 scores: (A) 95 yeast extracellular proteins (YEP) dataset; (B) 109 *K. lactis *predict extracellular proteins; (C) 95 *K. lactis *ramdom sequences (KLRS) from genome; (D) EPMS dataset from Swain *et al ***[22]** (E) Multivariate tests using Hotelling T^2 ^to verify the statistical significance**.

Using a statistical approach, the first GPS criteria, the signal peptide, was tested against the following datasets: YEP (yeast extracellular proteins sequences), KLRS (*K. lactis *random sequences), the predicted extracellular proteins determined by WoLF PSORT, and EPMS dataset by Swain *et al *[22] (Figure 2). The YEP scores showed NN S/D greater than 0.66 and HMM around 0.8, whereas KLRS simultaneously presented scores below 0.4 and 0.3 (Figs. 2A and 2C). The comparison between the controls for SignalP and the secreted ORFs scores revealed that the scores of the 109 ORFs were very similar to YEP, specifically, NN S/D was 0.56 and HMM was 0.78 (Figure 2B). Thus, the standards criteria provided by SignalP were correctly encountered in all 95 sequences from the positive control. The EPMS dataset showed a high NN score (>0.8) and high HMM score (>0.75) in 67% of sequences (Figure 2D). Although the other 33% were not detected as secreted by the predicted algorithms, the remaining 67% had a 0.74 probability of being equal to the predicted dataset according to T-square test (Figure 2E).

To evaluate the criteria for predicting the presence or absence of N-terminal signal peptides in the *K. lactis *dataset, the Hotelling T-square multivariate test (Figure 2E) was employed on the basis of NN Mean S/D and HMM scores. The vector parameters for each control set were compared to the predicted set and confirmed by T-square test. The estimated 109 ORFs were closer to the YEP dataset (p = 0.9401) than the KLRS (p < 0.01).

### Analysis of annotations

**Figure 3:**
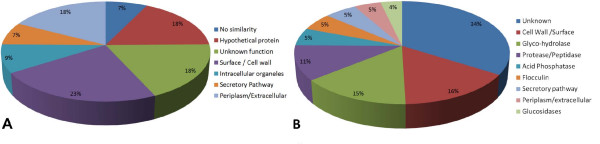
**Characterization of the predicted proteins from (A) subcellular location in Génolevures annotation (release 3) and (B) by function and conserved domains in Protein Family (PFam 23.0)**.

The biological significance of the predicted extracellular proteins of *K lactis *was determined on the basis of annotations available at the Genolevures website . Of the 109 predicted *K. lactis *extracellular proteins, 85 were annotated as similar to *S. cerevisiae*, and five as documented *K. lactis *proteins. Enzymes were the largest functional group (48%) of known predicted proteins. A smaller group (4%) was predicted as having a pheromone or mating-type function. Among the known sequences, 9% were considered intracellular proteins or wrong predictions (Figure 3A). For those unknown potential *K. lactis *extracellular proteins (25%), the Protein Family database (PFam) was applied to attempt to find relationships to known protein families through conserved domains (Figure 3B). The results demonstrated nine singletons among 21 that harbored conserved domains with varying PFam scores. The alpha mating factor precursor N-terminus (KLLA0A00154g, KLLA0F00220g), kappa casein (KLLA0B05731g), NADH dehydrogenase subunit 2 C-terminus (KLLA0C10054g), bacterial regulatory protein-Fis family (KLLA0D00660g), thioredoxin (KLLA0E05544g), mucin-like glycoprotein (KLLA0E10967g, KLLA0E19657g), and collagen triple helix repeat (KLLA0F01595g) all gave higher PFam scores. Analysis of the improbable secreted domains was carried out by alignment using BLAST tools . From nine sequences, six with nonsecreted domains were found to have a possible relation to extracellular proteins in other taxons.

### Relationship between the predicted extracellular proteins and transcriptional factors repertoire

The putative promoter region was taken as one kb upstream of each predicted extracellular protein-encoding ORF and analyzed using the Yeastract website tool [23] to identify TFBSs related to *S. cerevisiae*. The results indicated the presence of 65 different TFBSs. In addition, the supporting algorithms in Supplementary Materials found 23 TFs homologs in *K. lactis *when comparing the *K. lactis *TFs dataset published by Bussereau *et al *[18] to *S. cerevisiae*. At least two TFBS in each promoter region were established by this analysis. In the Yeastract database, all the TFs have Gene Ontology (GO) terms , that is, known details about the cellular function and address. These data (Table 1) showed that all 109 sequences have the TFBS for Mot3p (involved in repression of a subset of hypoxic genes and repression of ergosterol biosynthetic genes), 100 had a site for Stb5p (activator of multidrug resistance genes), 97 for Fkh1p (a minor role in the expression of G2/M-specific transcription in mitotic cell cycle), 45 for Gcn4p (activator of aa biosynthetic genes in response to aa starvation), 40 for Hac1p (regulates the unfolded protein response), 35 for Mcm1p (pheromone response), 33 for Rgt1p (regulates expression of several HXT glucose transporter genes in response to glucose), 28 for Nrg1p (mediates glucose repression and negatively regulates a variety of processes including filamentous growth and alkaline pH response), 21 for Adr1p (peroxisomal protein genes and genes required for ethanol, glycerol, and fatty acid utilization), 20 for Pho4p (phosphorylation at multiple sites and by phosphate availability), and 06 for Yap1p (required for oxidative stress tolerance; mediates resistance to cadmium). From this analysis, the TFs dataset is estimated to be a group that us likely to have a major influence on the extracellular secretome.

**Table 1:** Cluster of transcription factors with GeneOntology terms related to the predicted ORFs

Biological Process	T.F.	ORFs	Description Yeastract/GO
Aerobic/Anaerobic and Sterol metabolism	Mot3p	109	Repression of hypoxic genes, several DAN/TIR genes during aerobic growth, and ergosterol biosynthetic genes
	
	Hap4p	2	Subunit of the heme-activated, glucose-repressed Hap2p/3p/4p/5p CCAAT-binding complex, a transcriptional activator and global regulator of respiratory gene expression; provides the principal activation function of the complex

Cell Cycle	Fkh1p	97	The expression of G2/M phase genes; negatively regulates transcriptional elongation; positive role in chromatin silencing at HML and HMR.
	
	Cbf1p	11	Required for nucleosome positioning at this motif; targets Isw1p to DNA
	
	Ace2p	9	Activates expression of early G1-specific genes, localizes to daughter cell nuclei after cytokinesis and delays G1 progression in daughters.
	
	Rlm1p	5	Maintenance of cell integrity; phosphorylated and activated by the MAP-kinase Slt2p
	
	Swi5p	9	Transcription factor that activates transcription of genes expressed at the M/G1 phase boundary and in G1 phase

Drugs and metal resistance	Stb5p	100	Activator of multidrug resistance genes, forms a heterodimer with Pdr1p; interacts with a PDRE (pleotropic drug resistance element)
	
	Yap1p	6	Required for oxidative stress tolerance; activated by H2O2; mediates resistance to cadmium
	
	Yrr1p	7	Activates genes involved in multidrug resistance; paralog of Yrm1p, acting on an overlapping set of target genes

General stress response	Hac1p	40	Regulates the unfolded protein response, via UPRE binding, and membrane biogenesis; ER stress-induced splicing pathway utilizing Ire1p, Trl1p and Ada5p facilitates efficient Hac1p synthesis
	
	Gis1p	20	JmjC domain-containing histone demethylase; transcription factor involved in the expression of genes during nutrient limitation; also involved in the negative regulation of DPP1 and PHR1
	
	Msn2p	17	Transcriptional activator related to Msn4p; activated in stress conditions, which results in translocation from the cytoplasm to the nucleus; binds DNA at stress response elements of responsive genes, inducing gene expression
	
	Rtg3p	82	Basic helix-loop-helix-leucine zipper (bHLH/Zip) transcription factor that forms a complex with another bHLH/Zip protein, Rtg1p, to activate the retrograde (RTG) and TOR pathways (1, 2)

Pheromone response	Mcm1p	35	Involved in cell-type-specific transcription and pheromone response; plays a central role in the formation of both repressor and activator complexes.

Amino acid/Nitrogen starvation response	Gcn4p	45	Amino acid biosynthetic genes in response to amino acid starvation; expression is tightly regulated at both the transcriptional and translational levels
	
	Met4p	6	Responsible for the regulation of the sulfur amino acid pathway, requires different combinations of the auxiliary factors Cbf1p, Met28p, Met31p and Met32p

Carbon source response	Rgt1p	33	Glucose-responsive transcription factor that regulates expression of several glucose transporter (HXT) genes in response to glucose; transcriptional activator and repressor
	
	Adr1p	21	Required for transcription of the glucose-repressed gene ADH2, of peroxisomal protein genes, and of genes required for ethanol, glycerol, and fatty acid utilization
	
	Azf1p	15	Involved in induction of CLN3 transcription in response to glucose; genetic and physical interactions indicate a possible role in mitochondrial transcription or genome maintenance

pH stress response	Nrg1p	28	Recruits the Cyc8p-Tup1p complex to promoters; mediates glucose repression and negatively regulates a variety of processes including filamentous growth and alkaline pH response

Phosphate response	Pho4p	20	Binds cooperatively with Pho2p to the PHO5 promoter; function is regulated by phosphorylation at multiple sites and by phosphate availability

DNA Damage	Rph1p	17	JmjC domain-containing histone demethylase which can specifically demethylate H3K36 tri- and dimethyl modification states; transcriptional repressor of PHR1; Rph1p phosphorylation during DNA damage is under control of the MEC1-RAD53 pathway

**Figure 4:**
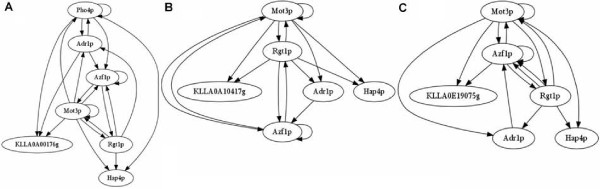
**Spanning trees of the carbon and aerobic response of the predicted transcriptional regulatory networks of (A) acid phosphatase precursor (KLLA0A00176g) (B) Invertase (KLLA0E19017); (C) α-factor mating pheromone (KLLA0E19075g); Transcriptional factors are represented by the small ellipses and target ORFs by larger ellipse**. The edges are the presence of TFBSs in putative promoter region.

The relationship between the transcriptional regulators and predicted extracellular proteome has great complexity. Therefore, to create an *ab initio *model, the data were shaped by graph theory. One of the graph representations was a square-directed non-weighted adjacency matrix, with 134 rows and columns. Among them, 109 were the predicted proteins identified in this study, 25 with their related TFs. The graph was created with 134 nodes and 884 edges. As illustrated in Figure 4, a three-spanning tree was extracted to illustrate the complexity of the regulatory network for each predicted ORFs. Three well-known extracellular proteins in *K. lactis *were use, along with α-factor mating pheromone (KLLA0E19075g), invertase (KLLA0A10417g), and acid phosphatase precursor (KLLA0A00176g). Supporting material can be found at our website, .

## Discussion

Because of its distinctive physiological properties, *K. lactis *has become an important model as a non-*Saccharomyces *yeast. In addition, *K. lactis *has great potential for biotechnological applications including expression of heterologous proteins [2]. These possibilities motivated us to study the global extracellular proteome and correlate it to TFs using a bioinformatics approach. The final results have shown 109 proteins that are potentially secreted by *K. lactis*. In addition to using the TMHMM and TargetP algorithsm used by Lee *et al *[10] and Swaim *et al *[22], the Phobius [19] and WoLF PSORT [21] were applied to find transmembrane domains and subcellular addressing that would direct targeted proteins to organelles such as the endoplasmic reticulum, golgi, and proteasomes. The WoLF PSORT algorithm appeared to be more accurate; also, when the dataset of secreted proteins detected experimentally by Swain *et al *[22] was compared by the predicting methods of Lee *et al *[10], it has detected more proteins (37) than WoLF PSORT (33). However, analysis of the prediction error rate was 69.3% for WoLF PSORT and about 79.2% for TargetP. The appearance of proteins in the medium changes in different physiological conditions [24], so the predictive methods chosen here decrease error rates and improve the chances of obtaining an actual extracellular protein in a given physiological condition. The error reduction may come from the incremented algorithmic Phobius [19] combining transmembrane topology and signal peptide prediction, and the new algorithm WoLF PSORT [21] to predict the subcellular localization of proteins on the basis of their amino acid sequences using *k*-NN (k-nearest neighbor). As described by Swain *et al *[22], in the signal peptide detection step, the prediction algorithm SignalP v3.0 [11] was used to give two NN prediction scores, mean S and mean D, and one HMM score. These NN scores were used for statistical analysis in the first step to identify extracellular proteins by the conserved secretory pathway features of a signal peptide and a signal peptidase cleavage site [10]. Accuracy in identifying extracellular proteins may be decreased because proteins that act in the periplasmic space or the cell wall also pass through the GPS. Motifs or conserved addresses for the perisplasmic space or cell wall have not yet been found. Thus, the strategy adopted to classify the results in this study focused on annotation terms and on PFam, a database of conserved domains and families [25]. The Genolevure third release is the main publicly available annotation dataset for *K. lactis *sequences. Therefore, the PFam [25] database was used in addition to updating the Genolevures annotation. Both showed five *K. lactis *annotated secreted proteins: acid phosphatase, repressible acid phosphatase precursor, guanosine diphosphatase, exo-1,3-beta-glucanase and invertase. Although some of these proteins have not been described as acting in the extracellular space according to Domínguez *et al *[26], *S. cerevisiae *proteins are not found free in the extracellular medium but are retained in the periplasmic space or associated with the cell wall. *K. lactis*, however, does not seem to have the same characteristic; in fact, it has been reported to secrete high molecular weight proteins [1]. Thus, in this study, proteins from the periplasmic space or associated with the cell wall have been considered as part of the potential extracellular proteins dataset.

Bioinformatics identifications are probabilistic in nature, so the advantage of our analysis lies in the low cost and high speed with which these identifications can be obtained [27,28]; hence, this analysis exploited an *ab initio *model of physiological inference. The model was created using the computational extracellular proteome dataset, the transcriptional regulators repertoire mined by Bussereau *et al *[18], and the Yeastract methodology created by Teixeira *et al *[23]. Since gene expression programs depend on recognition of specific promoter sequences by transcriptional regulatory proteins [18], we decided to analyze the relationship between the consensus sequences or DNA binding motifs and transcriptional regulators. One of the first changes that occurs in a cell after an environmental stimulus is the content of transcriptional regulators [24]. When a set of *S. cerevisiae *transcriptional regulators orthologues and their related DNA motifs binding sites was identified, a high level of polymorphism, or DNA binding factors capable of binding to both specific and nonspecific sequences, was observed [23,29]. Because of the complex relation between TFs and the predicted secretome, the data obtained was analyzed using graph theory [24]. The empirical model may suggest many conditions that have not yet been thought of by intuitive inference. The GO terms described for each TF dataset showed possible major interactions related to stress and the cell cycle. The results of this study are in accordance with the literature, because expressions of extracellular proteins increase in stress situations or in the exponential phase when the cell requires proteins that interact in the cell wall or in the periplasmic space [1]. However, for a good secretion system, a few different proteins that can show high expression and secretion are needed. An *ab initio *model allows searching for both these proteins and the environmental conditions that might improve their expression and secretion.

## Conclusion

Based on selected algorithms SignalP v3, Phobius, bigPI-predictor and Wolf PSORT, and adopting the highest Wolf PSORT *k-NN *scores and using multivariate T-square analysis for verification, we predicted an extracellular *K. lactis *secretome of 109 proteins. The well-known extracellular *K. lactis *proteins such as α-factor mating pheromone, invertase, and acid phosphatase precursor were among the 109 predicted proteins. In addition, by considering the Genolevure annotations and comparing to PFam, 48% of the known proteins had enzyme activity. By applying the *S. cerevisiae *Yeastract database, 65 transcription factor orthologues were found, 23 of which had binding sites in the promoters of the 109 predicted *K. lactis *secretome. An *ab initio *model of physiological inference is presented. The model is a graph with 134 nodes and 884 edges that suggests a large number of relationships between the proteins and physiological conditions that can be experimentally validated. Most of the predicted TF for extracellular proteins are related to stress responses, such as drug, acid and heat resistance, as well as nitrogen limitation, which may prove useful for inducing maximal expression of the potential extracellular proteins. A condition that favors secretion could be used to design a system to improve the secretion of a desired protein. our model is stored in a public database .

## Methods

### Data Sets

The main dataset analyzed in this study was in two files in FASTA format. Both files contained 5076 *K. lactis *nucleotide and aa sequences. These data are available in the *K. lactis *third public release from the Génolevures consortium .

To test the criteria for extracellular proteins, a validation set consisting of 95 non-redundant yeast extracellular proteins sequences (YEP) and 95 nonredundant *K. lactis *random sequences (KLRS) was assembled. The YEP dataset was obtained by searching in the UniProt protein database . The KLRS was assembled using a random number generator and a sequence seeker algorithm. Another validation dataset was manually extracted from Swain *et al *[22], consisting of 81 *K. lactis *extracellular proteins identified by mass spectrometry analysis (EPMS).

The *K. lactis *TF dataset used in this study was from Bussereau *et al *[18]. The retrieved data were composed of 102 TFs identified as orthologues of *S. cerevisiae *transactivators.

### Algorithms and Strategy

The entire *K. lactis *predicted proteins dataset was applied to SignalP v3.0 [11] to identify N-terminal signal peptides. To define a positive SignalP hit, the following simultaneous criteria were used: (a) signal peptide predicted by SignalP NN with the scores mean S and mean D; (b) signal peptide predicted by SignalP HMM considering the value of probability, and (c) signal peptide cleavage site located 10–40 aa from the N-terminus.

The group of predicted ORFs that encoded sequences with N-terminal signal peptides was analyzed according to the three additional characteristics of transmembrane domain, GPI modification site predicted by Phobius [19], and PI-predictor [20]; the subcellular location was estimated using WoLF PSORT [21] to identify signal addressing for subcellular locations. The obtained dataset comprised all sequences of deduced proteins potentially acting in extracellular space. The outcome set was analyzed by the PFam database [25] in order to update the Genolevure annotations.

To correlate the computational extracellular proteome and the TF repertoire, a supporting algorithm was created on the basis of ANSI/ISO C++ strings operations [30] in the *K. lactis *chromosomes dataset. This retrieved one kb upstream sequence as the putative promoter region from each predicted extracellular ORF. The recovered dataset is stored in a FASTA file with the relevant identification. The relationship between this computational extracellular proteome and the transcriptional regulators repertoire was made according to Yeastract [23]. The Yeastract web tools  and database were used to find associated TFBSs in *S. cerevisiae*. A second supporting C++ [30] algorithm was created to remove *S. cerevisiae *TFs nonhomologous to *K. lactis*. The Graphviz (Graph Visualization Software, ) package was used to draw the graph, and the spanning trees operations were implemented by Boost library 1.36 .

### Statistical Analysis

Multivariate analysis of variance was applied to verify the accuracy and determine the error rate of the computational secretome. The SignalP NN scores (mean S and D) and SignalP HMM probability were used as values in statistical analysis to determine the matrices of variance-covariance of the predicted and validations sets, and the Hotelling T^2 ^multivariate test [31] was applied to calculate the probability of equality of the means vectors.

## Authors' contributions

FP conceived and designed the project. OB developed and coded the support algorithms. LF analyzed the annotation of the transcriptional factors. CC performed the statistical analysis. All authors read and approved the final manuscript.
